# Comparison of Geniohyoid Muscle Morphology Assessment Using Conventional and Handheld Ultrasound Devices

**DOI:** 10.7759/cureus.94164

**Published:** 2025-10-08

**Authors:** Tetsuo Ota, Mai Sano, Mitsugu Yoneda

**Affiliations:** 1 Faculty of Health Sciences, Kanazawa University, Kanazawa, JPN; 2 Department of Rehabilitation, Hakuai Memorial Hospital, Tokushima, JPN

**Keywords:** geniohyoid muscle, handheld ultrasound device, morphological evaluation, relative and absolute reliability, swallowing, ultrasound imaging system

## Abstract

Background: Point-of-care ultrasound has recently gained attention for enabling simple and immediate bedside assessments of swallowing function. In particular, handheld ultrasound devices (HHUDs) are more suitable than conventional ultrasound imaging systems (UISs) in community and home settings due to their portability. The geniohyoid muscle (GM) plays a crucial role in swallowing by contributing to hyolaryngeal elevation, and its dysfunction has been associated with aspiration risk. Therefore, ultrasound assessment of the GM provides clinically important information for evaluating swallowing function. This study aimed to evaluate GM morphology using a UIS and an HHUD and to investigate the relative and absolute reliability, as well as the correlation between the two devices.

Methods: GM morphology was assessed using a UIS and an HHUD by measuring the longitudinal diameter, longitudinal cross-sectional area, transverse vertical diameter, transverse lateral diameter, transverse cross-sectional area, and contraction percentage. Relative reliability was calculated using intraclass correlation coefficients (ICCs(1,3)), while absolute reliability was evaluated via Bland-Altman analysis to detect fixed and proportional bias. Pearson correlation coefficients and paired t-tests were used to compare measurements between devices.

Results: High intrarater reliability (ICC > 0.9) was observed for all parameters except the contraction percentage. Bland-Altman analysis indicated fixed bias in longitudinal and transverse measurements. All parameters showed significant positive correlations between the UIS and HHUD, although the HHUD consistently yielded significantly smaller values.

Conclusion: HHUDs can be used for GM morphological assessment; however, clinicians should account for their tendency to produce smaller values compared to UISs. HHUD-based evaluation of the GM may have future applications in assessing sarcopenia-related dysphagia in older adults and in home-based clinical settings.

## Introduction

Eating is essential for human health. The swallowing function, which forms the basis of this activity, can easily decline with cerebrovascular disease, neurodegenerative disorders, and aging, leading to aspiration and reduced dietary diversity [1]. As the global population ages, the incidence and mortality of pneumonia, particularly aspiration pneumonia, are increasing. Aspiration pneumonia has been reported to be associated with longer hospital stays, higher mortality rates, and poorer prognoses compared to other types of pneumonia [2]. Given these severe outcomes, early and simple screening of swallowing function is critical to identify individuals at risk. Accordingly, several screening methods have been developed and are currently used, such as the Repetitive Saliva Swallowing Test [3], the Water Swallowing Test [4], and the Eating Assessment Tool-10 questionnaire [5]. For more detailed evaluations, gold-standard assessments such as the fiberoptic endoscopic evaluation of swallowing (FEES) and videofluoroscopic swallowing study (VFSS) are used [6]. However, both the FEES and VFSS are invasive, with radiation exposure in the VFSS and nasal discomfort or pain during probe insertion in the FEES. In addition, these examinations are typically limited to specialized medical institutions. Therefore, there is a growing need for the development of a novel, simple, and non-invasive method to assess swallowing function-especially for community-dwelling older adults.

In recent years, ultrasonography has gained attention as a non-invasive tool capable of visualizing internal features in real time. In applications related to swallowing function, it has been widely applied for various purposes, including anatomical evaluation of swallowing-related muscles (SRMs) [7], assessment of aspiration and pharyngeal residue risk [8], and evaluation of the effectiveness of swallowing training [9]. In addition, point-of-care ultrasound (POCUS), which can be performed immediately in clinical settings, has gained increasing attention. In the assessment of swallowing function, the miniaturization of ultrasound imaging systems (UISs) has made them more portable, facilitating their use not only at the bedside in hospitals but also in primary care settings such as community-based healthcare [10]. Handheld ultrasound devices (HHUDs) are often cited as alternatives to conventional UISs, offering several advantages, including lower cost, portability, and flexibility in terms of use location [11]. Their bedside application has been shown to improve diagnostic accuracy [12], reduce the number of additional diagnostic tests required [13], and enhance patient satisfaction [14]. However, HHUDs typically have lower spatial and temporal resolution compared to UISs, and their image quality is generally less clear. Therefore, it remains unclear whether HHUDs can achieve the same level of diagnostic accuracy as UISs [11]. To date, no studies have investigated whether HHUDs can be used to evaluate swallowing dynamics or the morphology of individual SRMs.

Swallowing is a complex process that involves the coordinated activity of multiple cranial nerves and oropharyngeal muscles and is typically divided into oral, pharyngeal, and esophageal phases. This neuromuscular coordination ensures safe and efficient bolus transport while protecting the airway. The geniohyoid muscle (GM), a key SRM, is involved in the anterior and superior movement of the hyoid bone during swallowing and in the relaxation of the upper esophageal sphincter [15]. Muscle atrophy or dysfunction of the GM has been associated with increased risks of sarcopenia, reduced tongue pressure, and aspiration [16]. Previous studies have demonstrated that ultrasound can be used to assess GM morphology and function, such as muscle cross-sectional area and contraction during swallowing, and have highlighted its potential utility for evaluating swallowing impairments [17,18]. Establishing a simple, portable method for evaluating GM morphology using HHUDs could facilitate the widespread assessment of SRM morphology and dynamics, ranging from bedside evaluations in clinical settings to use in community-dwelling older adults. Therefore, this study aimed to clarify the relative and absolute reliability, as well as the correlation, of GM morphology assessments using both UISs and HHUDs, thereby providing preliminary evidence for the potential use of handheld devices in swallowing muscle assessment.

## Materials and methods

Participants

This study was approved by the Medical Ethics Committee of our institution (No. 1032-1) and conducted according to the Declaration of Helsinki. Participants were recruited through an announcement at our affiliated institution between August 2023 and February 2024. Informed consent was obtained from all 37 healthy volunteers who participated in the study. We included healthy young adults without a history of cervical or oral diseases. The exclusion criteria were a history of cervical or oral diseases or any other condition that could interfere with swallowing function. To calculate the sample size for the intra-rater reliability assessment, we assumed an expected intraclass correlation coefficient (ICC) of 0.85, a minimum acceptable ICC of 0.70, a significance level of 0.05, a power of 80%, and three repetitions per participant, which indicated that at least 37 cases were required. In addition, an a priori power analysis (paired t-test, two-tailed) with an assumed medium effect size (d = 0.5), an alpha level of 0.05, a power of 0.80, and a correlation between measures set at 0.5 showed that a minimum of 34 participants was required to compare the two measurement methods.

Morphological assessment

**Figure 1:**
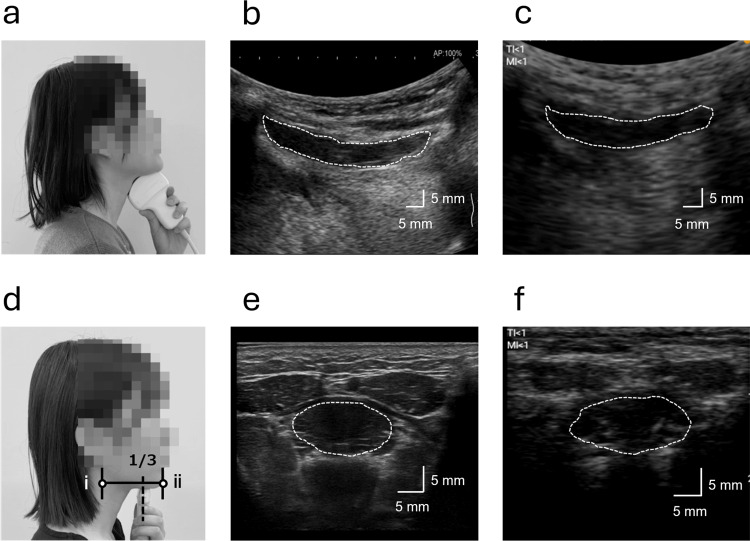
Ultrasound measurement and B-mode imaging of the geniohyoid muscle (GM) The regions enclosed by white dotted lines in the B-mode images represent the GM muscle belly. (a) Ultrasound measurement in the midsagittal plane. The longitudinal cross-section of the GM was recorded. (b) B-mode image obtained using the ultrasound imaging system (UIS) in the midsagittal plane. (c) B-mode image obtained using the handheld ultrasound device (HHUD) in the midsagittal plane. (d) Ultrasound measurement in the coronal plane. The measurement site was located approximately one-third of the distance from the anterior (ⅰ) to the posterior (ⅱ) margin of the mandible. (e) B-mode image obtained using the UIS in the coronal plane. (f) B-mode image obtained using the HHUD in the coronal plane.

Morphological assessments of the GM were performed using both a UIS (ARIETTA 750; FUJIFILM Corporation, Tokyo, Japan) and an HHUD (miruco; Nippon Sigmax Co., Ltd., Tokyo, Japan). A convex array transducer (ARIETTA 750: 1-5 MHz; miruco: 3.5 MHz) and a linear array transducer (ARIETTA 750: 5-18 MHz; miruco: 10 MHz) were used. The GM was measured in the submandibular midsagittal plane and vertically to the coronal plane of the mandible and in the upper one-third of the anterior and posterior ends of the mandible (Figures 1a, 1d). The convex array transducer was used to measure the longitudinal GM in the median sagittal plane, while the linear array transducer was used to measure the transverse GM in the coronal plane. B-mode imaging was used for all assessments, with images obtained from both the UIS (Figures 1b, 1e) and HHUD (Figures 1c, 1f), and all participants were seated upright during measurements. To ensure that the convex array transducer was placed directly against the skin, the participants were instructed to extend their necks slightly for the median sagittal plane measurement. The gain settings for the UIS were 60 dB in both the midsagittal and coronal planes, while those for the HHUD were 30 dB in the midsagittal plane and 50 dB in the coronal plane. To minimize potential order effects, the sequence of UIS and HHUD measurements was counterbalanced, such that approximately half the participants were assessed with UIS first and the others with HHUD first. The examiner was an occupational therapist specializing in rehabilitation, with 4-5 years of experience using UIS and HHUD.

**Figure 2:**
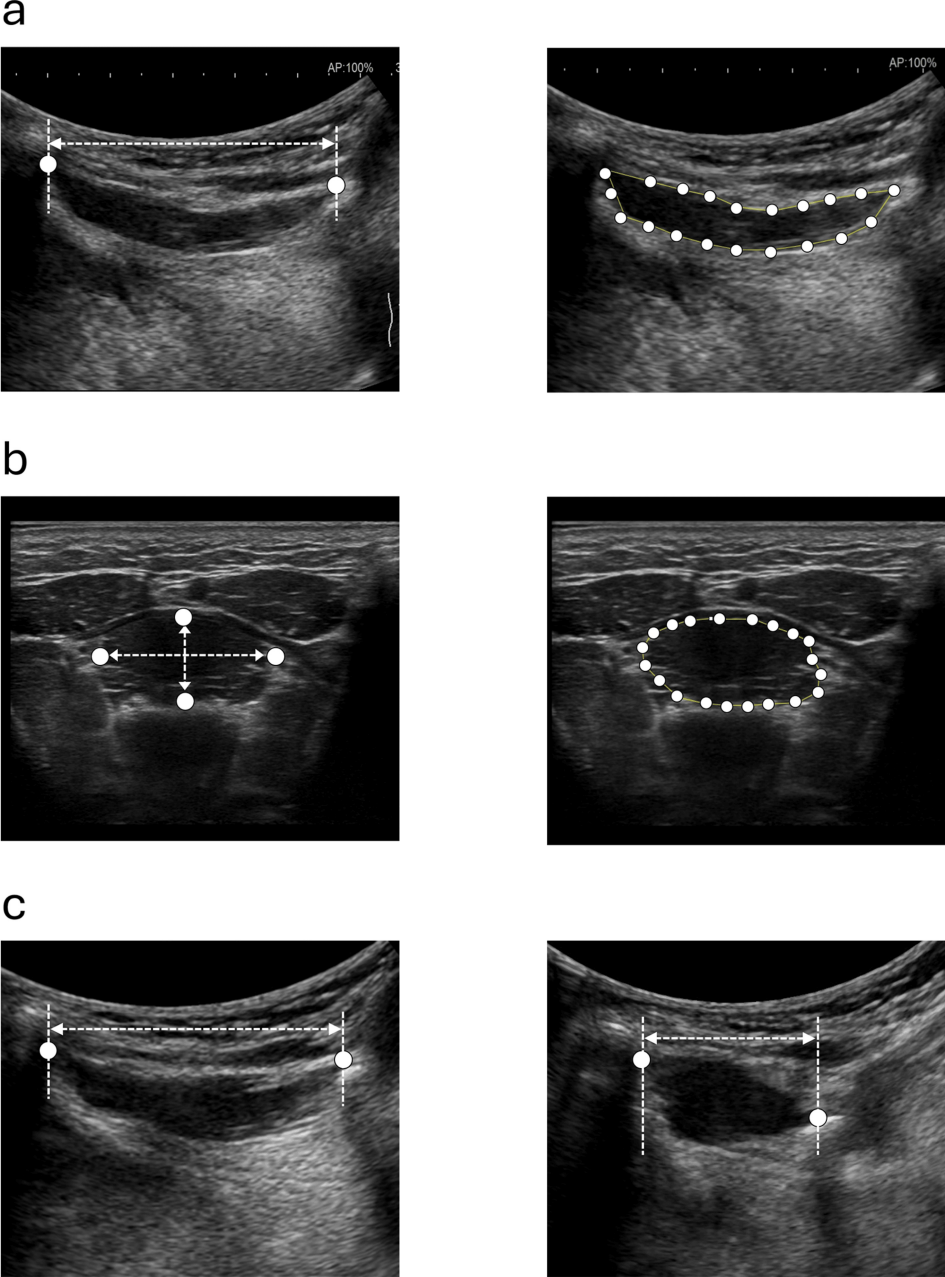
Morphological measurement parameters of the geniohyoid muscle (GM) Six parameters were measured using both the ultrasound imaging system (UIS) and handheld ultrasound device (HHUD) following the same procedures. (a) Longitudinal diameter was defined as the distance from the anterior to the posterior margin of the GM (left). Longitudinal cross-sectional area was calculated as the area enclosed by 20 evenly spaced points manually placed along the muscle belly margin (right). (b) Transverse vertical diameter was defined as the distance from the superior to the inferior margin of the GM (left), while transverse lateral diameter was the distance from the left to the right margin (left). The transverse cross-sectional area was measured as the area enclosed by 20 evenly spaced points placed along the muscle margin (right). (c) Contraction percentage of the GM was calculated from the resting and contracted diameters obtained in the midsagittal plane during swallowing.

Six different parameters were evaluated for the GM using both the UIS and HHUD: longitudinal diameter, transverse vertical diameter, lateral vertical diameter, longitudinal cross-sectional area, transverse cross-sectional area, and GM contraction percentage while swallowing (Figure 2). These parameters, except for the contraction percentage, were calculated from three still B-mode images. For the GM contraction percentage, participants were instructed to perform three consecutive dry swallows under standardized instructions. The movement of the GM in the midsagittal plane was recorded as a video file (30 frames per second (FPS) for the UIS, 8 FPS for the HHUD). For each swallow, the frames corresponding to the resting state (before swallow onset, when the GM was fully relaxed and elongated) and maximal contraction (during swallowing, when the GM reached its shortest length in the longitudinal plane) were identified. The GM contraction percentage was calculated using the following formula:

$\text{Contraction Percentage (\%)}=\frac{\text{Resting Diameter}-\text{Contracted Diameter}}{\text{Resting Diameter}}\times 100$

In four of the 37 participants (all male), the laryngeal prominence of the thyroid cartilage interfered with the transducer during swallowing, preventing it from being properly positioned against the submandibular region. Consequently, the contraction percentage could not be measured in these cases. All parameters were derived from the saved still images using ImageJ version 1.54 (https://imagej.net/ij/index.html), and the average value from three measurements was used for analysis. All images were analyzed by one experienced examiner without blinding to participant identity. Analyses were performed according to a prespecified protocol (fixed probe landmarks and identical display settings).

Data analysis

The normality of all parameters was assessed using the Shapiro-Wilk test. To examine the relative reliability between the UIS and HHUD, ICCs were calculated. Intra-rater reliability for three repeated measurements by the same examiner was assessed using ICC(1,3). This model is a one-way random-effects model with the average of three trials, which is appropriate when repeated measures are obtained from the same examiner without modeling examiner effects. The reliability was interpreted as follows: >0.90, great; >0.80, good; >0.70, fair; >0.60, possible; and <0.60, rework [19]. Absolute reliability was assessed by calculating the standard error of measurement and minimal detectable change at the 95% confidence level. Fixed and proportional biases were examined using Bland-Altman analysis. Fixed bias was considered to be present when the 95% confidence interval (CI) of the mean difference of measurements (DIFF) between UIS and HHUD measurements did not include zero. To examine the presence of proportional bias, Pearson’s correlation analysis was performed between the DIFF and the mean of the paired measurements. A Bland-Altman plot was constructed to visually assess data distribution. The plot displayed the mean difference of the DIFF along with the upper and lower limits of agreement (LoA). The LoA was calculated using the following formula: LoA = mean difference ± 1.96 × standard deviation of the DIFF. In addition, comparisons between the UIS and HHUD for each parameter were performed using Pearson’s correlation analysis and paired t-tests.

We estimated the effect size using Cohen's d statistic, with values of 0.20, 0.50, and 0.80 indicating small, medium, and large effect sizes, respectively. All analyses were performed using SPSS version 29 (IBM Corp., Armonk, NY, USA), with the significance level set at p < 0.05.

## Results

Participant characteristics

A total of 37 healthy volunteers participated in this study, consisting of 15 men and 22 women. The mean age was 21.5 ± 1.5 years, with an age range of 21-27 years.

Relative and absolute reliability for the UIS and HHUD

Table 1 presents the reliability of the UIS and HHUD in the morphological evaluation. Regarding relative reliability, intra-rater reliability for all parameters except the contraction percentage showed “great” ICC(1,3) values (0.91-0.96). In contrast, the contraction percentage showed poor reliability, with ICC(1,3) values of 0.54 for the UIS and 0.47 for the HHUD, classified as “rework.”

**Table 1:** Intra-rater reliability of each measurement UIS: ultrasound imaging system; HHUD: handheld ultrasound device; CI: confidence interval; SEM: standard error of measurement; MDC: minimal detectable change; ICC: intraclass correlation coefficient

Measurement direction	Parameter	Device	ICC(1,3)	95% CI	SEM	MDC_95_	Bland–Altman analysis			
							Fixed bias		Proportional bias	
							Mean difference	95% CI of difference	Correlation coefficient (r)	p-value
Longitudinal	Diameter (mm)	UIS	0.93	0.88–0.96	1.16	3.22	1.96	0.87–3.06	-0.06	0.74
		HHUD	0.95	0.92–0.97	0.99	2.74				
	Cross-sectional area (mm^2^)	UIS	0.95	0.91–0.97	12.57	34.83	3.54	-9.54–16.61	-0.05	0.77
		HHUD	0.96	0.94–0.98	10.51	29.14				
Transverse	Anteroposterior diameter (mm)	UIS	0.95	0.92–0.97	3.24	8.98	0.54	0.26–0.83	0.04	0.83
		HHUD	0.92	0.87–0.96	3.61	10.00				
	Mediolateral diameter (mm)	UIS	0.91	0.85–0.95	0.31	0.85	0.52	0.03–1.00	-0.05	0.77
		HHUD	0.91	0.84–0.95	0.38	1.04				
	Cross-sectional area (mm^2^)	UIS	0.95	0.92–0.97	0.62	1.71	13.77	7.58–19.97	0.13	0.45
		HHUD	0.92	0.86–0.96	0.67	1.85				
	Contraction percentage (%)	UIS	0.54	0.19–0.76	6.97	19.33	1.09	-0.82–2.99	-0.03	0.85
		HHUD	0.47	0.05–0.72	8.28	22.96				

**Figure 3:**
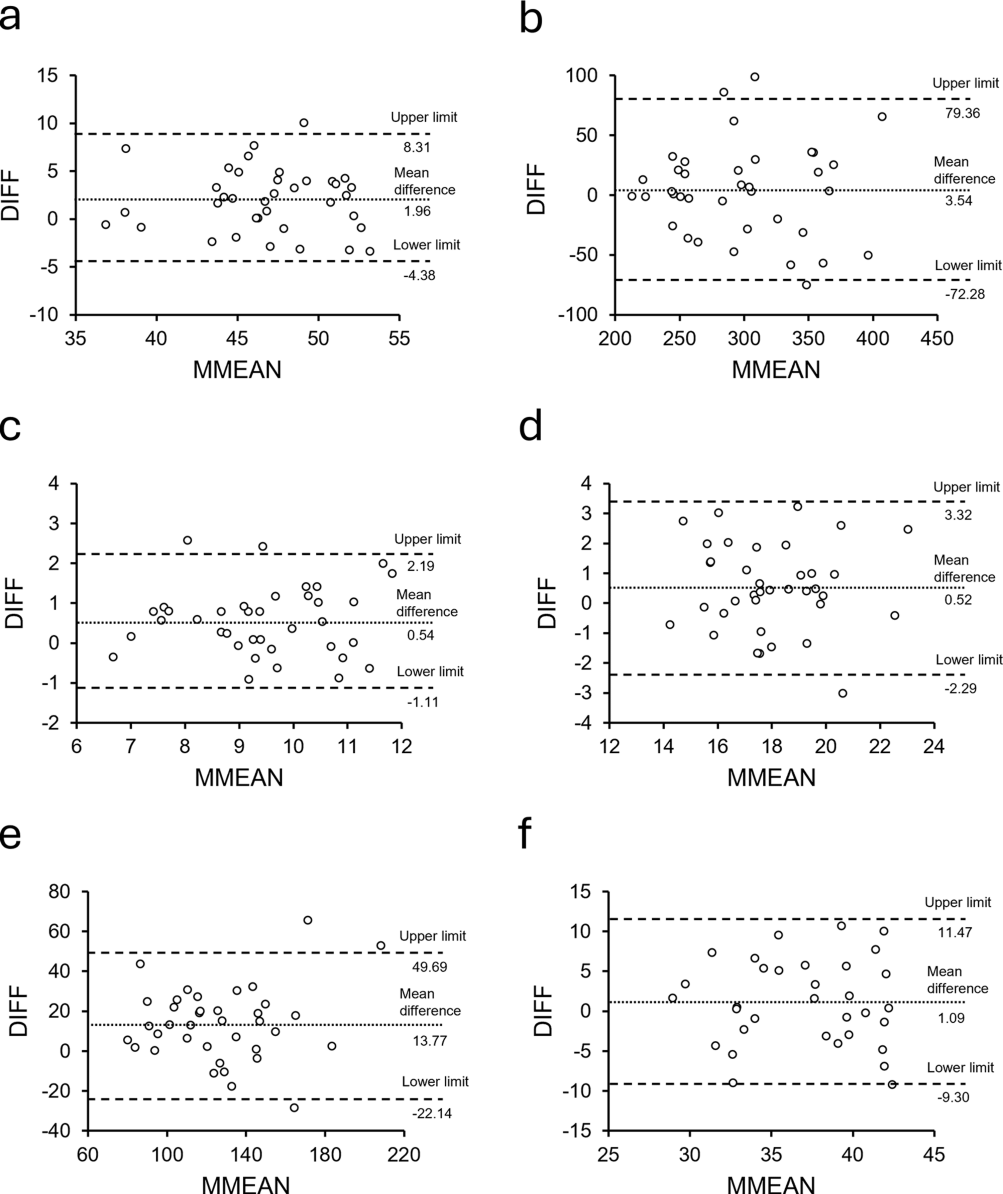
Bland-Altman plots for each measurement parameter The vertical axis represents the difference between ultrasound imaging system (UIS) and handheld ultrasound device (HHUD) measurements for each parameter (DIFF). The horizontal axis represents the mean of the UIS and HHUD measurements (MMEAN). In each plot, dashed lines indicate the mean difference (bias) and the upper and lower limits of agreement (LoA). (a) Longitudinal diameter; (b) longitudinal cross-sectional area; (c) transverse vertical diameter; (d) transverse lateral diameter; (e) transverse cross-sectional area; (f) contraction percentage.

For absolute reliability, Bland-Altman analysis revealed fixed bias in the longitudinal diameter, transverse vertical diameter, transverse lateral diameter, and transverse cross-sectional area, as the 95% CIs of the mean differences (DIFF) did not include zero. No proportional bias was observed in any of the parameters (Table 1, Figure 3).

Comparison and relationship between the UIS and HHUD

Table 2 presents the comparison between the UIS and HHUD. Significantly smaller values were observed with the HHUD compared to the UIS in the longitudinal diameter (t = 3.64, p < 0.01, d = 0.60), transverse vertical diameter (t = 3.87, p < 0.01, d = 0.64), transverse lateral diameter (t = 2.17, p = 0.04, d = 0.36), and transverse cross-sectional area (t = 4.51, p < 0.01, d = 0.74).

**Table 2:** Comparisons of each measurement by Student's t-test UIS: ultrasound imaging system; HHUD: handheld ultrasound device

Measurement direction	Parameter	Device	Value	Student's t	df	p-value	Cohen's d
Longitudinal	Diameter (mm)	UIS	47.89 ± 4.39	3.64	36	<0.01	0.60
		HHUD	45.92 ± 4.56				
	Cross-sectional area (mm^2^)	UIS	299.65 ± 53.58	0.55	36	0.59	0.09
		HHUD	296.11 ± 55.40				
Transverse	Anteroposterior diameter (mm)	UIS	9.73 ± 1.39	3.87	36	<0.01	0.64
		HHUD	9.19 ± 1.37				
	Mediolateral diameter (mm)	UIS	18.24 ± 2.09	2.17	36	0.04	0.36
		HHUD	17.73 ± 2.16				
	Cross-sectional area (mm^2^)	UIS	133.80 ± 31.50	4.51	36	<0.01	0.74
		HHUD	120.02 ± 29.29				
	Contraction percentage (%)	UIS	37.63 ± 4.78	1.16	32	0.26	0.20
		HHUD	36.55 ± 4.93				

**Figure 4:**
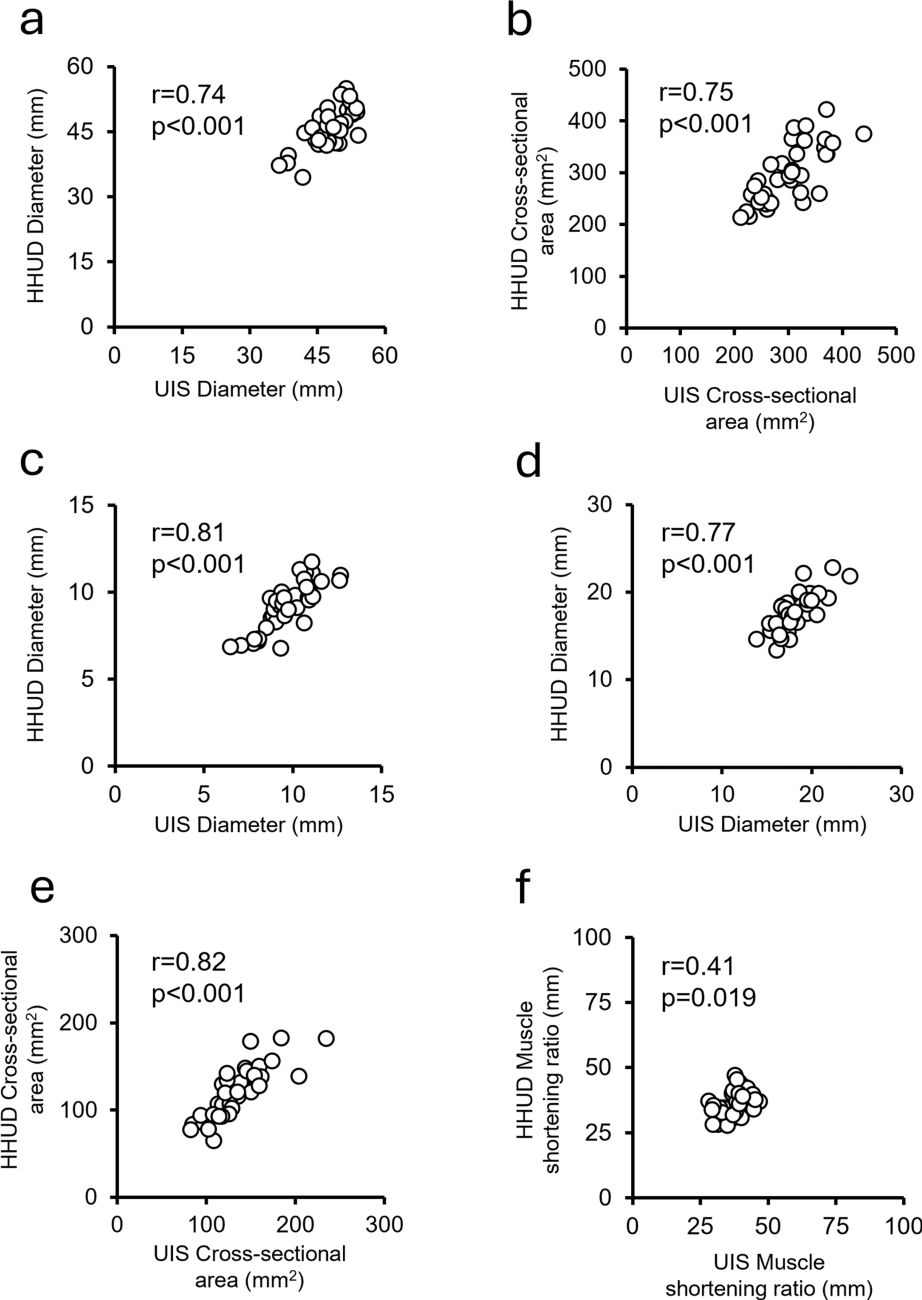
Correlation between UIS and HHUD measurements for each parameter Each scatter plot displays the correlation coefficient (r) and p-value, indicating the strength and significance of the relationship between measurements obtained from the ultrasound imaging system (UIS) and handheld ultrasound device (HHUD). (a) Longitudinal diameter; (b) longitudinal cross-sectional area; (c) transverse vertical diameter; (d) transverse lateral diameter; (e) transverse cross-sectional area; (f) contraction percentage.

Figure 4 shows the correlations between the UIS and HHUD. Significant positive correlations were found for all parameters: longitudinal diameter (r = 0.74, p < 0.01), longitudinal cross-sectional area (r = 0.75, p < 0.01), transverse vertical diameter (r = 0.81, p < 0.01), transverse lateral diameter (r = 0.77, p < 0.01), transverse cross-sectional area (r = 0.82, p < 0.001), and contraction percentage (r = 0.41, p = 0.02).

## Discussion

In this study, we investigated the relative and absolute reliability, as well as the correlation, between a UIS and an HHUD for the morphological evaluation of GM in healthy volunteers. The ICCs, representing relative reliability, showed "great" agreement for all parameters except the contraction percentage in both devices. In contrast, Bland-Altman analysis, representing absolute reliability, revealed fixed bias in the longitudinal diameter, transverse vertical diameter, transverse lateral diameter, and transverse cross-sectional area of the GM, with the HHUD producing significantly smaller values than the UIS. Taken together, these results suggest that GM morphology can be reliably assessed using either a UIS or an HHUD in healthy young adults. However, potential measurement discrepancies should be considered when comparing results across devices or when relating HHUD-based findings to those from previous UIS studies.

Relative reliability of GM morphological evaluation using the UIS and HHUD

The ICC(1,3) values obtained using the UIS and HHUD ranged from 0.91 to 0.96 for all parameters except the contraction percentage, indicating high relative reliability. These results are consistent with those of previous studies that reported high reliability in the morphological evaluation of SRMs using B-mode ultrasonography, including assessments of the tongue thickness, masseter muscle thickness, transverse cross-sectional area of the GM, and anterior belly of the digastric muscle [20,21]. These findings suggest that ultrasound-based morphological assessment of SRMs can be performed with high accuracy, regardless of device performance. In contrast, the contraction percentage of the GM, calculated from video recordings, showed ICC(1,3) values of 0.54 for the UIS and 0.47 for the HHUD, both classified as “rework.” Regarding GM activity during swallowing, Macrae et al. [17] reported intra-rater and inter-rater ICCs of 0.93 and 0.70, respectively, for the contraction percentage and 0.90 and 0.64 for the absolute change values. Although higher intra-rater ICCs were reported compared to the present study, their ICCs were calculated based on five repeated swallows performed at 30-second intervals. Similarly, Hsiao et al. [22] evaluated hyoid displacement induced by GM contraction during swallowing and reported intra-rater ICCs of 0.84 and 0.92 for two examiners and an inter-rater ICC of 0.81. Unlike the present study, which assessed GM contraction during three consecutive dry swallows, their study measured hyoid displacement during three swallows of 5 mL of water. Moreover, both of these studies included intervals between swallowing (30 seconds in Macrae et al.’s study and an unspecified duration in Hsiao et al.’s study), which may have contributed to the higher ICCs reported. In the present study, measurements were performed in the seated position without controlling for trunk or neck posture. Nevertheless, previous research has indicated that hyoid bone movement speed and distance during swallowing are not affected by posture [18]. To minimize potential bias, all measurements were performed by the same examiner, and the transducer placement was standardized to reduce variability. Although contraction percentage was calculated using a standardized swallow protocol, potential variability due to self-paced swallows and anatomical differences cannot be completely excluded. In addition, in four male participants, the prominent thyroid cartilage interfered with transducer placement, which may have limited the accurate assessment of GM contraction. Therefore, the “rework” classification observed for contraction percentage may have been influenced by the absence of intervals and the use of self-paced dry swallows by participants, and anatomical variations that hindered optimal transducer positioning. Impaired GM contraction can lead to reduced hyoid bone displacement, insufficient laryngeal elevation, and incomplete airway closure due to inadequate epiglottic inversion, making it a significant factor contributing to aspiration [23]. To facilitate the simple detection of aspiration risk at the bedside, it is necessary to explore more precise methods for measuring the GM contraction percentage.

Absolute reliability of GM morphological evaluation using the UIS and HHUD

Bland-Altman analysis revealed no proportional bias between the UIS and HHUD in the morphological assessment of the GM. However, fixed bias was observed in the longitudinal diameter, transverse vertical diameter, transverse lateral diameter, and transverse cross-sectional area, with the HHUD yielding significantly smaller values compared to the UIS. Comparative studies between UISs and HHUDs have been conducted in various medical fields, including internal medicine [24], respiratory diseases [25], rheumatic diseases [26], and cross-sectional area measurements of nerves [27]. The findings remain mixed, with some studies reporting that HHUDs provide diagnostic accuracy comparable to UISs [25,27], while others suggest that conventional UISs offer superior performance [24,26]. However, to the best of our knowledge, no previous studies have compared the performance of different ultrasound devices in the assessment of swallowing-related structures, making the present study the first to report such findings. In the present investigation, measurements obtained using the HHUD were smaller, and a fixed bias was identified. One possible explanation is the lower image quality of the HHUD compared to the UIS, which may have resulted in less distinct delineation of muscle boundaries (Figures 1c, 1f). Additionally, since GM morphology is influenced by factors such as age [28], sex [16,18], and body size [18], establishing standard reference values for the measured parameters remains challenging. Importantly, the presence of fixed bias has clinical implications. Because HHUDs consistently yielded smaller values than UISs, caution is required when interpreting absolute measurements or comparing results across different devices. While HHUDs may be suitable for tracking changes in the same patient over time, their application for establishing diagnostic thresholds or for direct comparison with reference values from UISs or other imaging modalities should take this fixed bias into account. Nevertheless, the findings of this study suggest that the use of HHUDs for evaluating GM morphology is feasible in both community healthcare settings and at the bedside and can be applied for both immediate and longitudinal assessments. However, results from HHUDs should be interpreted carefully when compared with those obtained from conventional UISs, computed tomography, magnetic resonance imaging, or other imaging modalities.

Future developments in research and clinical application

Traditionally, the assessment of swallowing function in community healthcare and bedside settings has relied on various screening tests [3-5] and subjective external observation and palpation [29]. The results of the present study suggest that objective evaluation of muscle morphology using an HHUD can be performed with high accuracy in such settings. If POCUS enables simple and accessible evaluation of GM morphology at the bedside or in community-dwelling older adults, it could facilitate not only immediate assessment of training effects but also the evaluation of dysphagia risk, such as sarcopenia-related dysphagia, without requiring hospital visits. Future studies should investigate whether an HHUD can also be used to assess the morphology of other SRMs, including the digastric, masseter, and infrahyoid muscles, as well as the presence or absence of aspiration in older adults [30], as was done in healthy individuals in the present study. If aspiration risk can be easily predicted through morphological assessment of SRMs using an HHUD before clinical symptoms appear, especially in older adults who traditionally seek care only after dysphagia develops, it may lead to the establishment of a new preventive approach that allows early intervention before the decline in swallowing function.

Limitations

This study has some limitations. First, the participants were limited to healthy young adults, and information on body size, such as height and weight, was not collected. Moreover, potential confounding factors such as age, sex, and obesity-related anatomical variations were not considered, which may have influenced the measurements. Future research should include older adults and individuals with dysphagia to compare the morphological characteristics of each group and should also examine whether body size influences these characteristics. Second, although the required sample size was met for most parameters, the contraction percentage analysis had a slightly smaller sample size due to participant exclusion, which may limit the statistical power for this specific parameter. Third, inter-rater measurements were not conducted. Although high intra-rater reliability was observed for all parameters except the contraction percentage, it remains necessary to examine whether similar reliability can be achieved between raters. Fourth, this study focused exclusively on the GM among the many SRMs. It is necessary to investigate whether an HHUD can also be used to reliably assess the morphology of other SRMs that play important roles in the swallowing process, such as the digastric, masseter, and infrahyoid muscles. Fifth, the findings of this study were obtained using a single HHUD model and, thus, cannot be generalized to other HHUDs not evaluated in this study. Further validation is needed to determine whether similar levels of reliability can be achieved with HHUDs of different specifications.

## Conclusions

The present study demonstrated that HHUDs can provide feasible and reliable assessments of GM morphology in young healthy adults, showing generally high agreement with conventional UIS. However, the limited sample, poor reliability of contraction percentage, and lack of inter-rater data warrant cautious interpretation and further validation in clinical and older populations.
